# Supplementary material for: Measuring Thousands of Single-Vesicle Leakage Events Reveals the Mode of Action of Antimicrobial Peptides
Source: Anal Chem. 2022 Jun 27;94(27):9530–9. doi: 10.1021/acs.analchem.1c03564 (PMC9280716; doi:10.1021/acs.analchem.1c03564)
Supplement: Supplementary file 1 — ac1c03564_si_001.pdf [file ac1c03564_si_001.pdf]

Supporting Information: Measuring thousands of single vesicle leakage events reveals the mode of action of antimicrobial peptides

Kareem Al Nahas,[†] Marcus Fletcher,[†] Katharine Hammond,[‡,¶] Christian Nehls,[§] Jehangir Cama,[†,‖,⊥] Maxim G Ryadnov,[*,‡,#] and Ulrich F Keyser[*,†]

†*Cavendish Laboratory, University of Cambridge, J.J. Thomson Avenue, Cambridge, CB3 0HE, UK*

‡*National Physical Laboratory, Hampton Road, Teddington, TW11 0LW, UK*

¶*London Centre for Nanotechnology, University College London, London, WC1H 0AH, UK*

§*Research Center Borstel, Leibniz Lung Center, Parkallee 10, Borstel, 23845, Germany*

‖*Living Systems Institute, University of Exeter, Stocker Road, Exeter, EX4 4QD, UK*

⊥*College of Engineering, Mathematics and Physical Sciences, University of Exeter, North Park Road, Exeter, EX4 4QF, UK*

#*Department of Physics, King's College London, Strand Lane, London, WC2R 2LS, UK*

E-mail: max.ryadnov@npl.co.uk; ufk20@cam.ac.uk

Contents

1 Microfluidic Chip Fabrication. **S-2**

2 Device Operation **S-4**

2.1 Vesicle Formation and Trapping S-4

2.2 Peptide Perfusion S-5

3 Data Acquisition and Analysis. **S-6**

4 Data Analysis and Filtering **S-6**

4.1 Analysis Procedure S-6

4.2 Description of Analysis S-7

4.3 Filtering S-8

5 Visualisation of Heterogeneity in Vesicle Populations **S-9**

6 Leakage Event Distributions for All Peptides **S-10**

References **S-16**

1 Microfluidic Chip Fabrication.

The GUV studio is a multilayered polydimethylsiloxane (PDMS) device that relies on standard photolithography and soft-lithography techniques.[S1] Three molds were fabricated matching the valve control layer (bottom), main chip (middle) and connector chip (top) as depicted in Figure 1. The main chip channels were molded from a master with two distinct channel profiles, (1) rectangular features with heights of ∼20 µm and ∼30 µm, and (2) rounded features with heights of ∼25 µm. The mold of the connector channel has features of ∼35 µm in height, whereas the features of the valve control layer are ∼30 µm in height. The SU-8 2025 negative photoresist was used for rectangular features with heights around 20, 30 and 35 µm

by spin-coating at 3800, 2800, and 2300 rpm respectively for 60 s with a ramp of 100 rpm s^{-1} on 4" silicon wafers (University Wafer, USA). The round channels required spin-coating AZ 40XT-11D positive photoresist at 2500 rpm on top of the SU-8 features. The spin coating was followed by a soft bake step where in the case of the negative resist, the coated wafer was placed on a hot plate for 1 min at 65°C and then 5 min at 95°C. In the case of the positive resist, the temperature was gradually increased from 80°C to 125°C over 7 min. A high-resolution, table-top laser direct imaging system (LPKF ProtoLaser LDI, Germany) was used for prototyping on the resist-coated substrates directly using AutoCAD designs. The mold then was post baked for 1 min at 65°C followed by 5 min at 95°C in the case of the negative resist, and 2 min at 100°C for the positive resist. The devices were developed in PGMEA for 3 min (SU-8) or AZ 726 MIF (AZ). Then the negative resists molds were hard-baked for 15 min at 125°C, and the positive resist mold was baked for $\sim$7 min at 120°C to re-flow the resist and obtain the round channels.[S1] To achieve different feature types and heights on the same silicon wafer, the photolithography process was performed three times on the same silicon wafer using a built-in anchoring tool for aligning the features.

PDMS replicas of the connector and main chips were obtained from the Silicon mold by mixing the elastomer with the curing agent (Sylgard 184, Dowsil) in a 9:1 ratio. The PDMS mixture was then degassed for 30 min in a desiccator, poured onto the molds and baked in the oven for 90 min at 60°C. The valve control PDMS membrane was obtained using a mixing ratio of 14:1 (elastomer:curing agent) and spin-coating the PDMS on the mold at 1500 rpm for 60 s with a ramp of 100 rpm s^{-1}. After curing and peeling off the main chip, the inlets/outlet were punched with a 0.75/1.5 mm biopsy punch (WPI, UK) respectively. A glass slide (76 mm × 38 mm × 1 mm) was coated with PDMS and used as the base for the devices. The main chip was aligned and bonded to the PDMS valve control membrane by exposing the surfaces to an air plasma (100 W plasma power, 10 s exposure, 25 sccm, plasma etcher from Diener Electric GmbH & Co. KG) and then annealing the exposed surfaces. After bonding, the main chip was peeled off and the valve control inlets

created using the 0.75 mm biopsy punch. The sample was then bonded to the PDMS-coated glass slide using air plasma. The surface of the microfluidic channels delivering the outer aqueous solution (OA) was treated for 15 min with polyvinyl alcohol (PVA) solution (50 mg mL^{-1}, 87-90% hydrolysed, Sigma-Aldrich).[S2,S3] Post treatment, the PVA was removed via vacuum pump suction and baked at 120°C for 15 min. Finally, the connector PDMS layer was aligned and plasma bonded on top of the main chip.

2 Device Operation

After assembly the chip is mounted and filled as previously described,[S3] briefly the fluids in the chip were controlled using 8 pressure ports and a single syringe pump module, 3 for the three OLA inlets (vesicle formation), 4 for the 8 perfusion inlets (each pressure port is split into two reservoirs using external fittings) and 1 for the 3 valves. After mounting the device, the first step is filling the 3 dead-end valve control channels with milliQ water. Later, the main flow channel is filled with OA solution from the main outlet (outlet A) in the direction of the outlet in the connector chip (outlet B). Shortly after the OLA reservoirs are connected and formation should start before the OA solution reaches the OLA outlet and traps any remaining air. Once an air-free device is obtained, the experiment can commence. A valve is actuated by applying ∼200-500 mbar.

2.1 Vesicle Formation and Trapping

Vesicle formation and trapping is initiated by actuating valve (V1) and applying suction at the lower outlet A (10 μL/h). The GUV stream diverges from being exclusively directed into outlet B and towards the traps. Actuating V1 allows the suction stream to disregard the perfusion inlet stream and dedicates the volumetric flow onto the GUV stream in the connector chip, in turn increasing trapping efficiency without increasing the flow rate in the traps. The flow rate at the entrance of each chamber during trapping was calculated to be

between 1 and 1.5 μL/h.

2.2 Peptide Perfusion

After vesicle entrapment and verifying traps are occupied with GUVs, valve 1 is relaxed, valve 2 is actuated and the suction tube is removed leaving outlet A open. This reduces the functioning platform circuitry to perfusion inlets and vesicle chambers, whilst excluding the OLA component and connector chip for the remainder of the experiment. The reservoir with the peptide mixture is exchanged with the connected IA reservoir for each chamber and the input pressure was adjusted to ∼10 mbar to drive the peptide solution into the chamber. During that process, the trapped GUVs are washed with the remaining IA buffer that predominates the tygon tubing and should amount to ∼32 μL. It was calculated that the application of ∼10 mbar at each perfusion inlet results in a flow rate at the entrance of each chamber of ∼10.7 μL/h. Acquisition is promptly initiated afterwards.

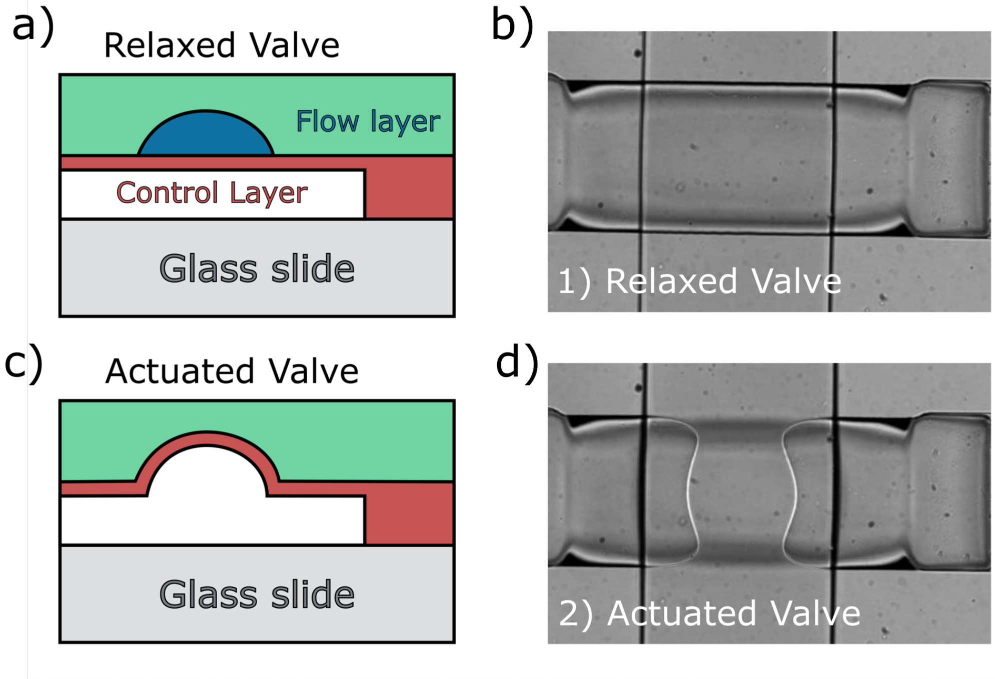

Figure S1: Schematic of microfluidic valve action. A thin layer of PDMS beneath the fluidic channel network may be actuated from "OFF" (a) to "ON" by the application of air pressure beneath the thin PDMS layer (c). b) and d) show images of the "OFF" and "ON" states respectively.

Figure S1 illustrates the action of the microfluidic valves. These enable dead volumes of fluid within the tubing interfacing fluid reservoirs and the GUV studio to be replaced to avoid large lag times during peptide perfusion.

3 Data Acquisition and Analysis.

The device was mounted on an Olympus IX73 inverted microscope equipped with an automated Prior XYZ-stage, a Photometrics Evolve 512 camera coupled with a wLS LED lamp (QImaging), a FITC filter cube set (Chroma) and a 10× air objective (Olympus UPLFLN). The software µManager 1.4 was used to synchronize the camera acquisition and the movement of the automated stage. Each chamber was designed such that it could be imaged within 8 fields of view (FOVs), hence an image acquisition cycle over the entire device consisted of 64 FOVs. The automated stage takes 1 min to scan all 64 FOVs. Vesicle monitoring continued overnight and we reported 450 cycles after peptide arrival in the GUV trap chambers. Vertical motion of the GUVs is restricted by the height of the microfluidic device to ensure consistent focal volumes throughout the experiment. The acquired videos were analysed using a bespoke automated python code to detect GUVs and obtain fluorescence signal traces for individual vesicles.

4 Data Analysis and Filtering

4.1 Analysis Procedure

All experiments were analysed using a custom made python software package, the source code for which we have made available on github. The software receives 64 experimental videos each corresponding to a different field of view (FOVs) covering the full network of traps. These FOVs are grouped such that 8 consecutive FOVs constitute one experimental chamber. Each experimental video is analysed in turn, outputting a csv file containing

intensity time traces for all GUVs identified. These csv files are collated to group GUV time traces by chamber. Subsequently, the GUV time traces within each chamber are ordered by the time at which a GUV Leakage Event occurs to produce heat plots as in Figure 3.

4.2 Description of Analysis

First the start of the experiment is automatically identified. This is achieved by including a small concentration of tracer dye in the peptide solution perfused in the experiment. To identify the arrival of the drug, the software calculates the median intensity of the field of view over the course of the full experimental video. This approximates the background fluorescence due to the tracer dye. The time at which the background reaches half its final intensity is defined as the start of the experiment, $t = 0$. To aid detection of GUVs they are detected at the time just before a significant increase in the background intensity is observed. Detection is performed by first creating a binary image in which all pixels are classified as either foreground, 1 or background, 0 by Otsu's thresholding method.[S4] Here we exploit the fact that before the background dye has arrived, there are only two intensity classes in our image, one bright and one dark. The dye within the GUVs are the only source of bright pixels and all other pixels are background. Therefore, after thresholding the binary image delimits GUV pixels from background. Next, segmenting individual GUVs in the binary image is achieved using a 'euclidean distance transform' (EDT) on the binary image and then a peak detection function, both from the scikit image python library.[S5] The EDT calculates the euclidean distance of each foreground pixel from the nearest background pixel and assigns this value to the given foreground pixel to create a transformed image. For roughly circular foreground objects, peaks occur in the EDT at the object's centre (given it has the furthest distance to its nearest background pixel). Thus finding such peaks allows us to find the centre coordinates of individual GUVs across the FOV. About these centre coordinates, 30 x 30 pixel Regions of Interest (ROIs) are taken forward for analysis. For each subsequent frame in the experimental video, the analysis loops over all ROIs first finding

pixels within GUVs by Otsu's method and thus the mean intensity over all pixels within a GUV. Eventually, after a LE, thresholding fails as the GUV intensity is no longer significantly greater than the background intensity. At this point the mean intensity across the whole ROI is recorded for the remainder of the experiment. This procedure stops after either 600 mins or the end of the experimental video, whichever is first. The final background intensity is determined by finding the average intensity of the final 5 frames of the experimental video. This is subtracted from each GUV intensity profile. The profile is then normalised by its own maximum intensity.

4.3 Filtering

Various filtering procedures are required to distinguish spurious intensity changes from real Leakage Events. Post-hoc filtering procedures are run at the end of the analysis routine. One such spurious intensity drop occurs when a GUV escapes its trap and as such the intensity recorded in the corresponding ROI drops between consecutive frames. When this occurs, predominantly, the GUV reappears in the next frame in a position downstream of its initial ROI. These instances are detected by the software and the relevant intensity time traces removed from the sample. Where the GUV completely leaves the FOV, it is not possible to distinguish these events from a bursting LE and therefore it is not removed. As well as this, GUVs sometimes enter ROIs after the leakage event of the original occupier. These events are also removed from the final sample. A GUI was created to allow human oversight at several steps in the analysis procedure. For example, the GUI allows the user to identify that appropriate ROIs are found, view the single GUVs within their ROIs over the full experimental time and verify that thresholding occurs correctly.

5 Visualisation of Heterogeneity in Vesicle Populations

Figure S2 displays the response of a sample of 5 GUVs exposed to R-NI01 dosage at 10 μM. The heterogeneity of the LE time points can be seen.

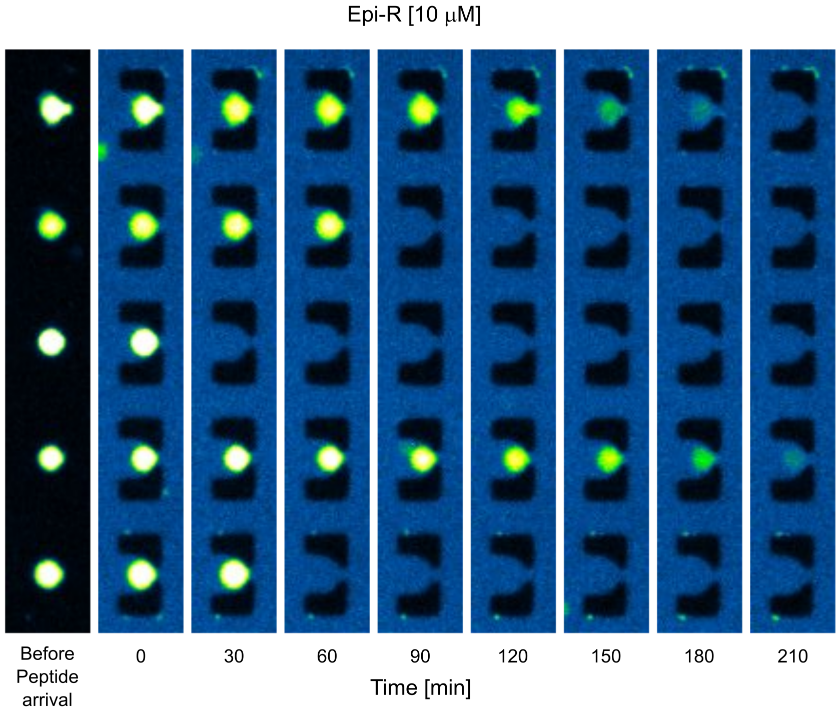

Figure S2: Sample of vesicles from the R-NI01 peptide experiment in which GUVs amongst the sample show heterogeneity in response to R-NI01 dosage.

6 Leakage Event Distributions for All Peptides

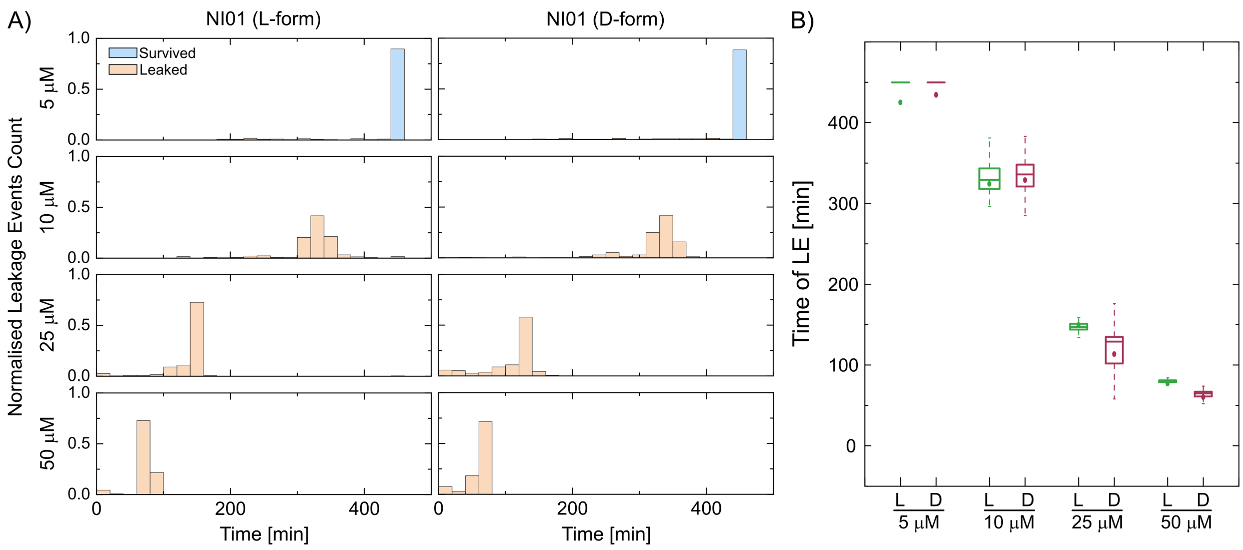

Figure S3: Side-by-side comparison between the all L- and all D- forms of NI01 using histograms and a box plot depicting the LEs time distributions normalized to the count of GUVs monitored. The histograms and box plot correspond to the heat maps for Figure 3 in the manuscript.

Table S1: Summary of leakage events mean time point and GUV population survival within 7.5 hours of peptide treatment with the all L- and all D- forms of NI01.

Peptide	Conc.	Average time	Std	Survival %
Epi N	5	433.7	51.4	89.6
Epi N	10	322.2	44.0	0
Epi N	25	136.2	34.6	0
Epi N	50	74.4	14.8	0
Epi N-D	5	432.4	54.8	88.5
Epi N-D	10	320.8	48.1	0
Epi N-D	25	109.2	37.9	0
Epi N-D	50	57.6	15.7	0

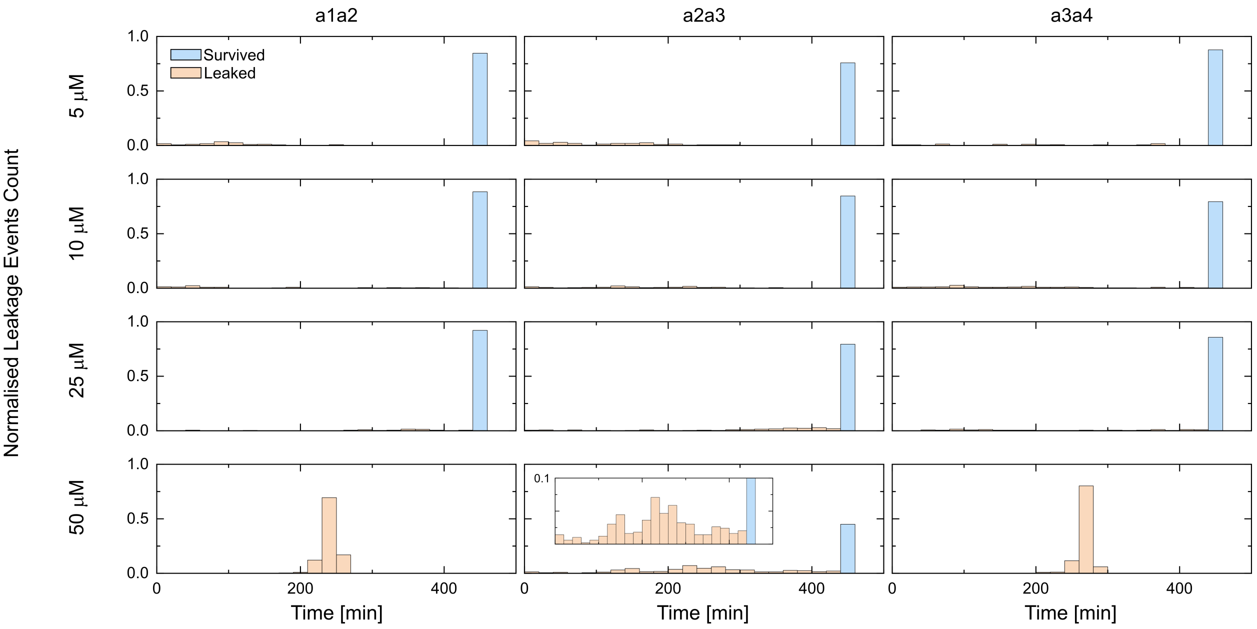

Figure S4: Side-by-side comparison between the three substructures of NI01 using histograms depicting the LEs time distributions normalized to the count of GUVs monitored. An inset was added for $\alpha2\alpha3$ at 50 µM to better visualize the wide distribution of LEs below 10% of the normalized count. The histograms correspond to the heat maps for Figure 4 in the manuscript.

Table S2: Summary of leakage events mean time point and GUV population survival within 7.5 hours of peptide treatment for the three substructures of NI01.

Peptide	Conc.	Average time	Std	Survival %
$\alpha1\alpha2$	5	396.7	126.4	84.6
$\alpha1\alpha2$	10	415.5	104.5	88.4
$\alpha1\alpha2$	25	437.4	49.4	92.2
$\alpha1\alpha2$	50	240.1	10.7	0
$\alpha2\alpha3$	5	368.0	150.1	75.8
$\alpha2\alpha3$	10	405.3	109.1	84.6
$\alpha2\alpha3$	25	419.5	79.9	79.3
$\alpha2\alpha3$	50	335.3	126.2	44.9
$\alpha3\alpha4$	5	390.5	125.1	87.7
$\alpha3\alpha4$	10	419.5	89.7	79.3
$\alpha3\alpha4$	25	417.9	90.5	85.7
$\alpha3\alpha4$	50	267.4	10.8	0

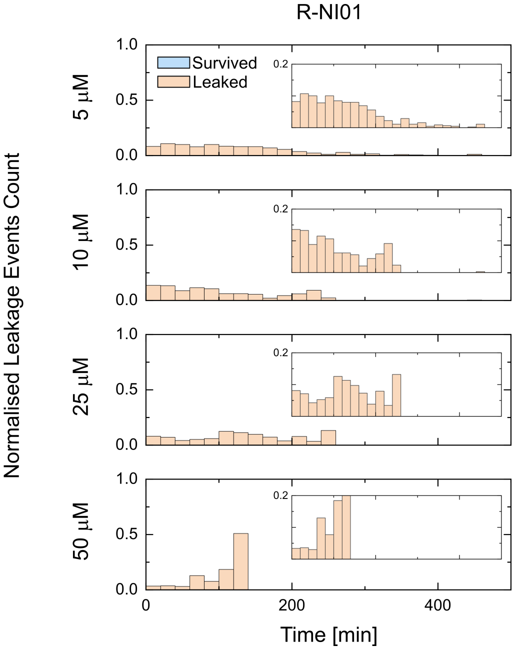

Figure S5: The time distribution of LEs induced by the mutant R-NI01 depicted in histograms normalized to the count of GUVs monitored. An inset was added for each concentration to better visualize the wide distribution of LEs below 20% of the normalized count. The histograms correspond to the heat maps for Figure 5 in the manuscript.

Table S3: Summary of leakage events mean time point and GUV population survival within 7.5 hours of peptide treatment with the mutant R-NI01.

Peptide	Conc.	Average time	Std	Survival %
Epi R	5	122.6	90.4	0
Epi R	10	102.8	76.2	0
Epi R	25	134.0	74.4	0
Epi R	50	103.0	32.3	0

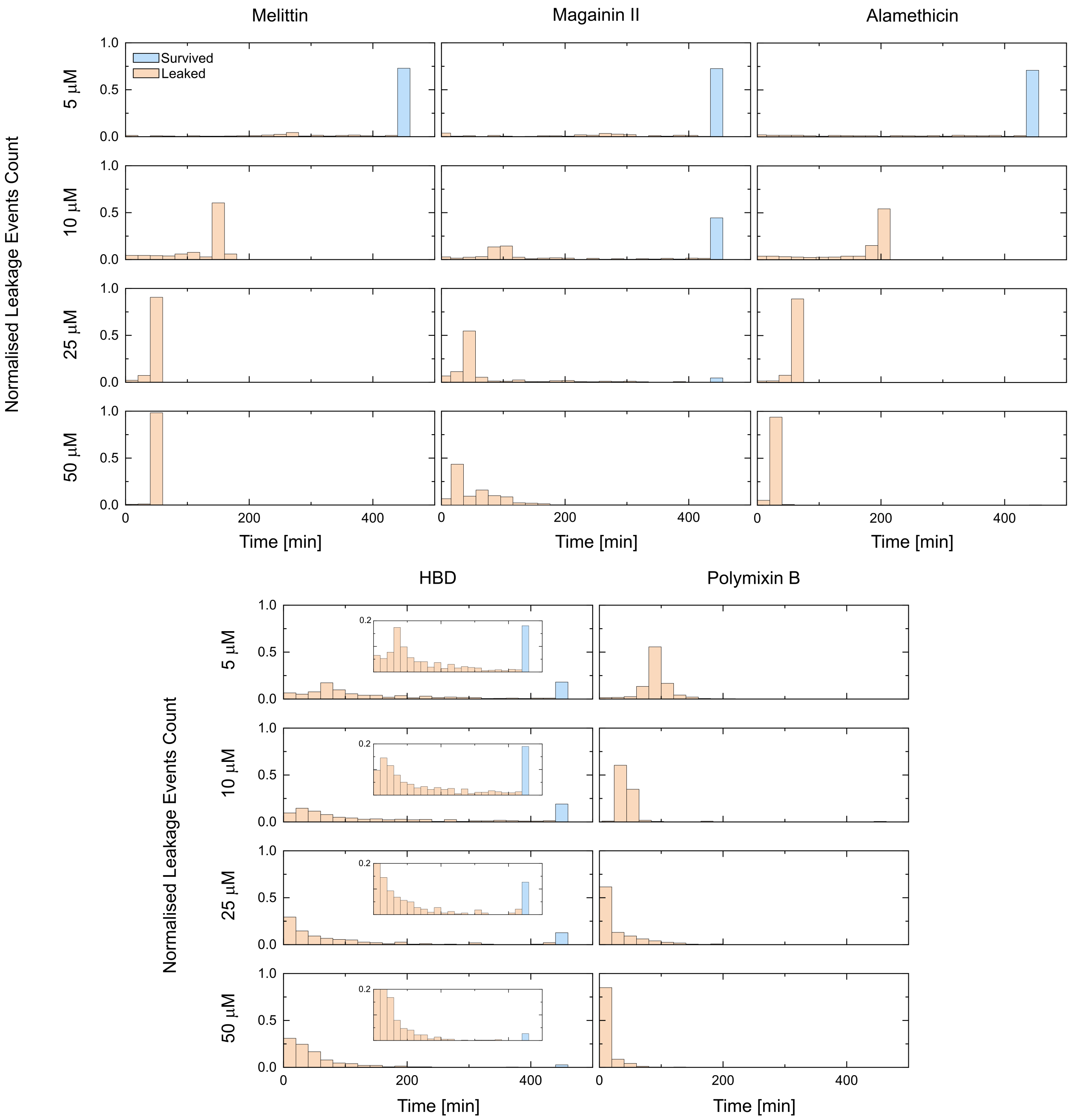

Figure S6: Side-by-side comparison between 5 archetypal AMPs of melittin, magainin 2, alamethicin, polymyxin B and hβD-3-l in anionic PC/PG (3:1) GUVs. Histograms depicting the LEs time distributions normalized to the count of GUVs monitored. An inset was added for each concentration for hβD-3 to better visualize the wide distribution of LEs below 20% of the normalized count. The histograms correspond to the heat maps for Figure 6 in the manuscript.

Table S4: Summary of leakage events mean time point and GUV population survival within 7.5 hours of peptide treatment with the 5 archetypal AMPs (melittin, magainin 2, alamethicin, polymyxin B and hβD-3-l).

Peptide	Conc.	Average time	Std	Survival %
Melittin	5	393.9	107.7	72.9
Melittin	10	124.9	44.9	0
Melittin	25	47.8	7.7	0
Melittin	50	50.0	4.4	0
Mag-2	5	384.5	124.1	72.3
Mag-2	10	274.7	173.9	44.3
Mag-2	25	85.8	108.2	4.5
Mag-2	50	50.7	37.6	0
Alam	5	376.6	133.9	70.9
Alam	10	165.3	62.6	0
Alam	25	61.0	17.9	0
Alam	50	34.1	23.8	0
Polym	5	92.9	33.0	0
Polym	10	47.1	34.9	0
Polym	25	28.6	35.9	0
Polym	50	11.5	16.9	0
hβD	5	182.3	153.4	18
hβD	10	179.9	164.4	18.9
hβD	25	123.6	153.0	12.6
hβD	50	59.9	82.6	2.69

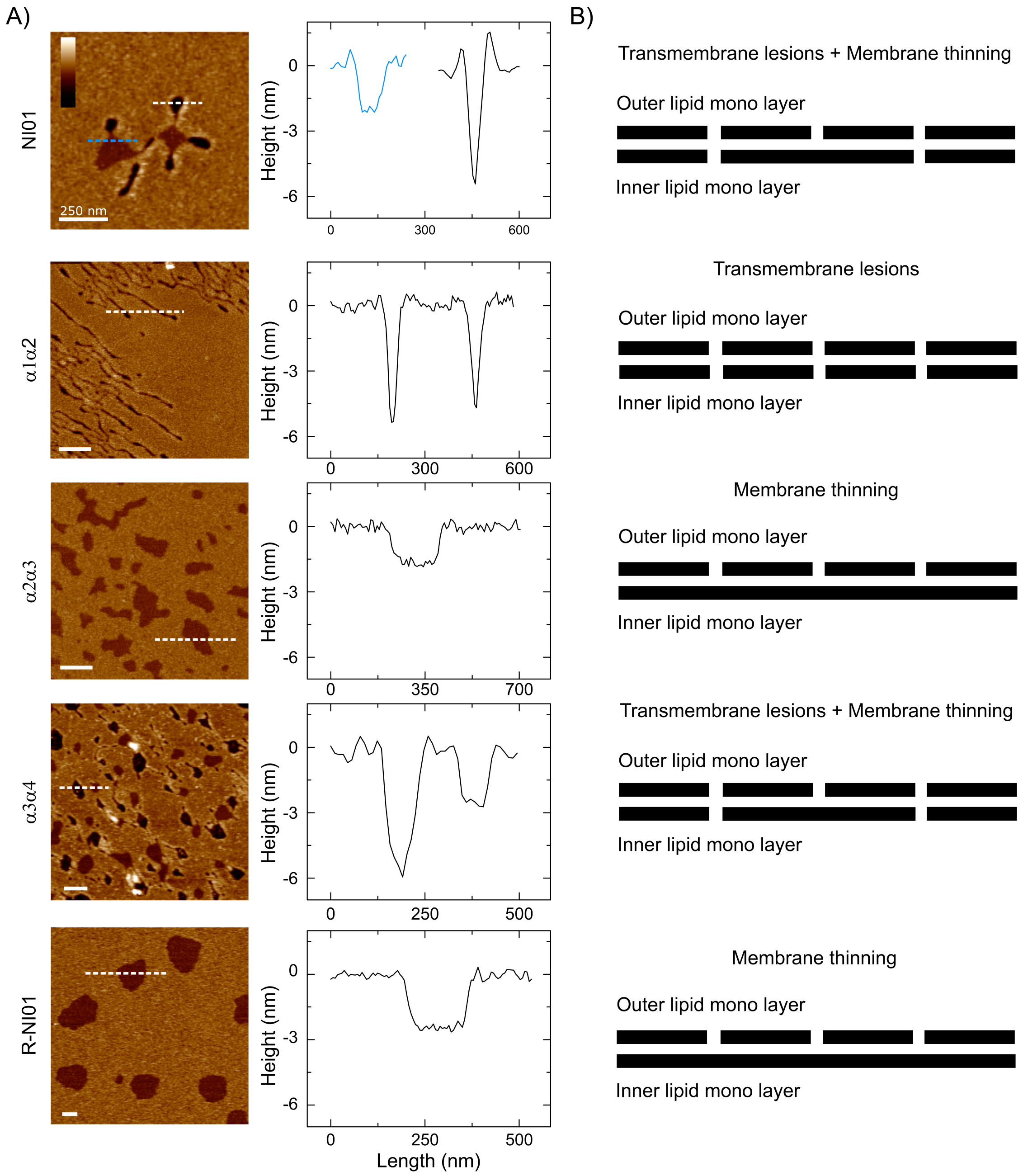

Figure S7: Outline for the suggested mechanisms of the bacteriocin-derived series of peptides. A) In-liquid AFM images representing the topography of PC/PG (3:1) SLBs mimicking bacterial membranes treated with NI01, three substructures derived from NI01 and the arginine mutant R-NI01. NI01 produces floral shaped topography that consists of bilayer spanning petal shaped transmembrane lesions (dark regions) and a membrane thinning patch in the center (light regions). B) Schematics representing the different mechanisms for each of the peptides as interpreted from the in-liquid AFM topography images.

References

(S1) van der Linden, A. J.; Yelleswarapu, M.; Pieters, P. A.; Swank, Z.; Huck, W. T.; Maerkl, S. J.; de Greef, T. F. A multilayer microfluidic platform for the conduction of prolonged cell-free gene expression. *J. Vis. Exp.* **2019**, *2019*, e59655.

(S2) Deshpande, S.; Dekker, C. On-chip microfluidic production of cell-sized liposomes. *Nat. Protoc.* **2018**, *13*, 856–874.

(S3) Al Nahas, K.; Cama, J.; Schaich, M.; Hammond, K.; Deshpande, S.; Dekker, C.; Ryadnov, M. G.; Keyser, U. F. A microfluidic platform for the characterisation of membrane active antimicrobials. *Lab Chip* **2019**, *19*, 837–844.

(S4) Otsu, N. A Threshold Selection Method from Gray-Level Histograms. *IEEE Transactions on Systems, Man, and Cybernetics* **1979**, *9*, 62–66.

(S5) van der Walt, S.; Schönberger, J. L.; Nunez-Iglesias, J.; Boulogne, F.; Warner, J. D.; Yager, N.; Gouillart, E.; Yu, T.; the scikit-image contributors, scikit-image: image processing in Python. *PeerJ* **2014**, *2*, e453.
